# Orchestrating a community-developed computational workshop and accompanying training materials

**DOI:** 10.12688/f1000research.16516.1

**Published:** 2018-10-17

**Authors:** Sean Davis, Marcel Ramos, Lori Shepherd, Nitesh Turaga, Ludwig Geistlinger, Martin T. Morgan, Benjamin Haibe-Kains, Levi Waldron

**Affiliations:** 1 Center for Cancer Research, National Cancer Institute, USA, Bethesda, MD, 20892, USA; 2 Graduate School of Public Health and Health Policy and the Institute for Implementation Science in Population Health, City University of New York, New York, NY, 10027, USA; 3 Roswell Park Cancer Institute, Buffalo, NY, 14203, USA; 4 Princess Margaret Cancer Centre, University Health Network, Canada, Toronto, Ontario, M5G1L7, Canada; 5 Medical Physics Department, University of Toronto, Toronto, Ontario, M5G1L7, Canada

**Keywords:** education, software, informatics, bioinformatics, Bioconductor, R, literate programming, markdown, github, open source

## Abstract

The importance of bioinformatics, computational biology, and data science in biomedical research continues to grow, driving a need for effective instruction and education. A workshop setting, with lectures and guided hands-on tutorials, is a common approach to teaching practical computational and analytical methods. Here, we detail the process we used to produce high-quality, community-authored educational materials that are available for public consumption and reuse. The coordinated efforts of 17 authors over 10 weeks resulted in 15 workshops available as a website and as a 388-page electronic book. We describe how we utilized cloud infrastructure, GitHub, and a literate programming approach to robustly deliver hands-on tutorials to participants of the annual Bioconductor conference. The scripts, raw and published workshop materials, and cloud machine image are all openly available. Our approach uses free services and software and can be adapted by workshop organizers and authors in other contests with appropriate technical backgrounds.

## Introduction

Methods of biomedical data analysis are rapidly evolving, creating a crucial need for constantly up-to-date learning materials. Workshops given by topical experts that combine didactic lectures with hands-on, guided tutorials are a common approach to teaching data analysis. The educational materials produced by such workshops, however, are often difficult to find or utilize by the rest of the community after the workshop is over. Each year, the Bioconductor project organizes and hosts a scientific conference that features scientific talks, poster presentations, networking sessions, and hands-on workshops. Nearly half of the conference time is devoted to hands-on workshops ranging from introductory to specialized and advanced topics. We sought an organized approach to developing workshop materials that meets the goals of allowing multi-disciplinary content by multiple contributors, providing “literate” code ^1^ that is presented in context with its explanation and runs reproducibly, can be disseminated broadly in a self-contained format, and can be efficiently updated for the next iteration. This article describes the approach adopted by the Bioconductor 2018 conference to coordinate 15 workshops, contributed by 17 authors, deliver these to conference participants, and then freely disseminate a book of the materials. The book is available at https://bioconductor.github.io/BiocWorkshops/.

With a relatively large number of contributors, we set out to produce workshop materials that:

1. Maintained a basic level of functionality and standardized style,
2. Could be used for interactive sessions and as standalone educational materials;
3. Could be improved by community contribution and input;
4. Could be formally published online *and* as a “published” work;
5. Would incorporate some educational best-practices;
6. Would promote smooth workshop offerings by allowing students to work on cloud instances known to run all workshops without error; and
7. Would allow easy re-use of the instructional materials and cloud instances by others after the conference.

The approach involved a call for workshop syllabi that were vetted by committee, and requiring that authors of accepted workshops contribute their materials in R Markdown format to a central GitHub repository. Workshop editors compiled the collated book with Bookdown ^2^, using Packer ( https://packer.io) to reproducibly create an Amazon Machine Image (AMI) capable of compiling the workshops. The AMI was also used to provide cloud-based virtual machines to participants during the workshops. By performing ongoing code testing on this same AMI, and using issue tracking to inform authors of issues with their code, workshop organizers ensured smooth delivery of workshops without software installation slowdowns or unexpected issues with incompatible computational environments. We describe the process in order to enable others to use the approach and the specific code we created, and to highlight the areas identified for further improvement. The process uses free services and software and can be replicated without monetary cost given appropriate technical background of authors and editors.

## Requisite technical skills

The process described requires at least one editor with the computational skills to:

1. Create a Bookdown project;
2. Test workshops and build the collated book;
3. Provide debugging help to workshop developers;
4. Use git branches and GitHub pages to publish the book; and
5. Create the virtual image containing all workshop materials and required software, ideally using Packer and/or Docker ( https://www.docker.com) for reproducibility.
6. Use GitHub issue labels and assignments to organize and assign tasks.

We expect that the workshop syllabus template, bookdown configuration files, build scripts, Packer definitions, and model GitHub repository may be helpful for other groups considering applying a similar approach to workshop creation.

Workshops authors must to be able to:

1. Author R Markdown document (or another literate programming style agreed on by the organizers) and supporting files; and
2. Make commits to GitHub.

The editors exempted one author from the third requirement, but this was for an already well-tested workshop. This would not be practical for other workshops that required iterations of debugging and testing. All other authors made their own commits to GitHub.

## Methods

### Making the call for workshops

The process began with a public call for workshop proposals in the form of a syllabus including a summary, a schedule, prerequisites, and learning goals. Requiring such a syllabus allowed i) the committee to carefully vet the instructional qualities of proposals, ii) participants to better anticipate the contents and learning outcomes of each workshop, including those needing to justify conference attendance in terms of specific learning objectives, iii) prompted instructors to devise learning goals and objectives for their workshops, and iv) promoted standardization of workshop formats. The syllabus proposal was also included at the start of each workshop and “chapter” of the produced book.

A template in Markdown format was provided with the call for syllabi (see http://bit.ly/2OfqlI9). The syllabus template included a minimal summary of relevant pedagogical theory to guide workshop authors in creating meaningful learning goals and objectives.

### Deadlines

For authors of accepted syllabi, deadlines were given for i) submission of a draft workshop, and ii) a complete workshop that compiles without errors. The first deadline, approximately 4 weeks before the conference, proved important to engaging authors at a sufficiently early stage to provide them with “collaborator” access to the repository and begin the sometimes lengthy process of debugging both individual workshops and the collection as a whole. The final deadline, 1 week before the conference, gave editors time to debug individual workshops with errors, build the conference workshop book, and create the cloud image to be used by conference participants. In hindsight, these deadlines seemed adequate provided that editors were available for an intensive period of engagement starting from just before the first deadline. In the end, 2 days after the final deadline were allowed for resolving errors, at which point the deadline was considered passed and access privileges of everyone but editors was changed to read-only. This process left 4 days for the editors to fix remaining overall and workshop-specific problems without new ones being introduced, successfully build the book of materials, review the book for more subtle problems like missing images and incorrect formatting, and create an AMI for use by participants at the workshop.

### Issue tracking

We used the GitHub Issue-tracking system to track the progress of individual workshops and to resolve project-wide issues. Authors were asked to submit a new issue as a way of providing their GitHub usernames, after which they were given collaborator write permission to the repository. A single issue was created for each accepted workshop and given the custom label “Not Started”, using the `ghi` GitHub Issue command-line tool ( https://github.com/stephencelis/ghi) to streamline creation and labeling of 15 new issues. This process could be streamlined by creating the per-workshop issues first, then asking authors to make a comment on the issue for their workshop. As workshop authoring progressed, the “Not Started” label was replaced with color-coded labels of “Incomplete,” “Problems,” “Looks OK,” and “Status Unknown.” Editors used these issues to document and share workshop-specific build errors, and authors used them to respond and ask workshop-specific debugging questions. We did not find the Travis Continuous Integration (CI) system ^3^ usable due to the resource intensiveness of building the workshops, and the need to test each workshop individually as well as the book as a whole. Automated testing of such a project, including posting of workshop-specific errors to the correct issue, is an area for future improvement.

### The testing and debugging cycle

We applied the approach of the Bioconductor project to ensure smooth-running workshops in which i) instructors could present a document containing runnable code and results, while ii) students could follow along by running the provided code, without wasting limited workshop time due to students running different software versions, having to install software and dependencies, or trying to run broken code. To accomplish this, we implemented the workshops in a development cycle characterized by rapid changes and continuous integration, followed by a release cycle frozen to new functionality. The development cycle included the initial phase of author contribution, an initial freeze of 3 days where authors were prevented from further contributions but editors continued debugging and building, and a final release freeze 2 days before the start of the workshop. The final freeze was used to resolve dependencies and to build and test the Cloud image used by conference participants. This schedule provided maximum development time to authors but required an intensive development period for organizers immediately before the conference, so we emphasize the importance of these final deadlines being “hard” ones.

At the start of the initial development phase we provided instructions in the README.md file of the project GitHub repo ( https://github.com/Bioconductor/BiocWorkshops) ^4^ on how authors could test their workshops individually. This testing procedure was a departure from procedures most authors were already familiar with, and some authors submitted untested code. In an ideal Continuous Integration environment, each new commit would have been automatically and centrally tested, but the editors resorted to a manual process of regularly testing the individual workshops and providing feedback. This was in part due to limitations of the free tier of most continuous integration systems. (e.g., https://docs.travis-ci.com/user/customizing-the-build/#build-timeouts). Considering some features of the BiocWorkshops repository, such as a 20 gigabyte repository (including cache and data files) and about 130 R package dependencies, it may be more practical to set up a paid or in-house Continuous Integration environment for future work. Another possibility is to build and test each workshop in an individual GitHub repository, with these being incorporated as Git submodules in a master repository ( https://git-scm.com/book/en/v2/Git-Tools-Submodules).

### Reproducible and robust workshop materials

Workshops were authored using R Markdown, and compiled into a book (PDF and ePub) and website using Bookdown R package. Bookdown, in turn, uses the gitbook publishing system ( https://www.gitbook.com/) to produce a variety of formats from the same source material. R Markdown files intended to be part of a Bookdown project do not contain the required front matter of a typical stand-alone R Markdown document. To help authors use and test the correct format, we seeded each workshop document with the syllabus that had been submitted by that author, and successfully built the book of the submitted syllabi. Each workshop represented a chapter of a book compiled using the Bookdown software. This approach provided several advantages:

- R markdown syntax is already familiar to any developer of a Bioconductor package, since it is the standard approach to creating the package “vignette” or prose documentation.
- R markdown implements “literate programming” by including formatted text, runnable code, and output of the code
- Bookdown allows collating chapters as a clean, lightweight online book format, and pandoc additionally allows creation of PDF and ePub formats
- These formats can then be self-published with options to order paper copies through companies such as https://leanpub.com

This approach allowed automatic installation of required packages by listing them in the DESCRIPTION file required by R packages.

### Organization, coordination, and editorial responsibilities

The organization and coordination process benefited from a long-term working relationship among editorial team members, prior experience with organizing similar conferences, and good communication among team members. Division of responsibilities was natural based on team member interests and skills. Additional deadlines or organizational structure might be required in cases where editors and organizers are less familiar with each other’s skills and interest.

The following editorial jobs were required. Except where noted as “committee”, each task was assigned to a single individual, clarifying responsibilities.

- Creating the syllabus template
- Reviewing and selecting submitted syllabi (committee)
- Reminding of upcoming deadlines, and chasing after authors who have missed deadlines
- Content scoping including:
  - Labeling workshops as “Learn”, “Apply”, or “Develop” (committee)
  - Advising some authors on overly lengthy or out-of-scope material
  - Cleaning up formatting such as lengthy automatically generated messages
  - Assigning course numbers to group similar workshops together
- Creating the publication scripts (Bookdown shell)
- Testing individual workshop documents and posting issues to GitHub (multiple editors)
- Central debugging of individual workshops when needed (multiple editors)
- Building the whole book and publishing it through GitHub-pages
- Creating a packer script to automate AMI creation
- Final AMI creation and cloning for the conference

### Delivering a common, convenient, efficient computational environment

We chose to use commercial cloud service (Amazon Web Services (AWS)) to provide stable networking, common hardware and software, and minimize venue IT requirements and installation problems during the workshops. An AMI is a template that can be used to launch many identical virtual machines. The Bioconductor project maintains AMIs for recent versions of R and Bioconductor ( http://bioconductor.org/help/bioconductor-cloud-ami/), which we extended iteratively throughout the workshop development and editing process. The final AMI, including RStudio server and R, required Bioconductor and R packages, and all workshop materials and dependencies, was used to launch an identical virtual machine for each workshop participant. Based on the ongoing testing, we knew that the image (AMI ID: ami-bac2c5c5) was adequate to run all workshop materials. The approximate cost of running all instances was less than $1000 for 5000 compute hours. The technology for distributing unique images to each workshop participant is an area for future improvement. For example, using Kubernetes ( https://kubernetes.io/) on a Cloud cluster to instantiate Docker images for each participant may be a more cost-effective and portable solution.

### Building the workshop AMI

Rather than manually installing new software or modifying virtual machine configuration by hand, we adopted the infrastructure-as-code paradigm using the Packer toolkit ( https://packer.io). The requirements to build the virtual machine image were described in a JSON format ( https://www.json.org/) file which Packer can use as input to reproducibly create an AMI.

The AMI was used by workshop organizers to build and test the workshop materials during the workshop editing process and was regenerated multiple times as individual workshop authors updated their respective materials and dependencies. In particular, with each addition of new R packages or dependencies, a new AMI was created.

The R package system already includes a well-defined approach for describing dependencies, the “DESCRIPTION” file ( https://cran.r-project.org/doc/manuals/r-release/R-exts.html#The-DESCRIPTION-file). We asked workshop contributors to make additions to a single DESCRIPTION file in the workshop git repository when adding or changing dependencies. The presence of a correctly formatted DESCRIPTION file is sufficient to comprise an R package, so including the DESCRIPTION file in the top-level directory of workshop materials allowed standard R installation mechanisms to add dependencies to each AMI version.

### Providing virtual machines to participants

After the finalized AMI was available, a custom application was used to launch a virtual machine for each participant using the AMI as a template. For this particular set of workshops, the machines were configured to launch an m4.xlarge instance (4 virtual CPUs, 16 GB of RAM) with 100 GB of disk storage. As the virtual machines were accessed via an Rstudio server, all participants shared a common user experience through the Rstudio interface that nearly all were already familiar with.

To gain access to a personal virtual machine with the workshop materials, each user supplied a user ID (the user’s email) and a password that was the same for all participants. A new virtual machine was then launched and the attached IP address associated with the user’s email address. Further attempts by the same user to launch a virtual machine would simply supply the participant with the same instance. The virtual machines were created using a common Bioconductor account and were terminated automatically after the conference.

## Results

The process organized 15 workshops presented at the Bioc2018 conference into a single publicly available book. Each workshop comprised a single self-standing chapter of the book. Instructors directly presented their chapter materials at the conference, potentially complemented by slides and live-coding. Workshop participants were able to run the workshop code without software installation or errors, making efficient use of the 1- or 2-hour workshop time for learning through both observation and application.

Workshop materials are available via a website, a PDF book, an ePub book, and the commercial self-publication service, https://leanpub.com. The PDF version includes 388 pages, the result of 19 contributors making 313 separate changes (commits) to the materials over the course of 10 weeks. Workshops were qualitatively classified as 100-level “Learn”, 200-level “Apply”, or 500-level “Develop”, with 4, 9, and 2 chapters, respectively. Whereas conference attendance was ~120 participants, approximately 10 times as many have viewed the published materials in the 2 months since they were posted online.

## Discussion

We have described the approach adopted by the Bioconductor 2018 conference organizers to coordinate 15 workshops, contributed by 17 authors, and deliver these to participants over 22 total hours of instruction and hands-on tutorials (see http://bioc2018.bioconductor.org/schedule). The process described above includes a combination of social contracts such a deadlines and clear responsibilities of organizers and contributors that, when combined with social coding practices and modern publishing tools, facilitated the creation of 388 pages of content (PDF version) over the course of 6 weeks in a format that can readily be updated annually. A workshop-specific AMI and virtual machines enhanced the participant experience by eliminating time-consuming installation problems and ensured that appropriate IT infrastructure (compute, storage, and performant networking) were available.

Bioconductor, as a project and as a community, values openness and sharing. Workshops were developed publicly with the goal of creating not only published materials, but having a set of modifiable and iterable raw material that others can repurpose for their own learning or instruction outside the context of the Bioconductor conference.

### Advantages gained by this approach

We were able to quickly develop a coherent and cohesive set of workshop materials that were appropriate for interactive sessions and as standalone educational materials. Providing a content template with specific required fields resulted in materials that could be quickly evaluated by potential participants with respect to i) included content, ii) learning objectives and goals, and iii) prerequisites. Using R markdown-based content ensured that included code would run without error. Cloud infrastructure minimized the need for local compute resources, limited network bandwidth needs to participant computers, and guaranteed an identical and tested compute environment. Clear deadlines and responsibilities for both contributors and editors resulted in 15 out of 16 proposed workshops to be included in the final materials, suggesting that contributors understood the requirements at the time of proposal and were able to follow through without our process introducing an undue burden.

### Author perspective

The workshops proved easy to create due to the set pattern of guidelines present for submitting a workshop. The guidelines helped instructors to think about the structure and time limitations of the workshop, and to communicate precise learning goals, providing little chance to skew off topic. The R markdown format is particularly helpful for reproducibly compiling and collating the workshops ahead of time, because this format is widely familiar in the Bioconductor community. This format is completely open source, and authors did not have the need to create new “accounts” or “pay” for any services to contribute to this format. All work happened on Github in a centralized open source environment, allowing authors to take technical and pedagogical insights from other workshop authors. Testing that an individual contributed workshop was compatible with the whole bookdown document was straightforward given the use of common tools in our community. This ease of local testing made producing the workshop quite efficient. It was important that each workshop author was responsible only for ensuring that her own content could build successfully, without concern for formatting or integration into the entire document and website.

### Generalizability to other programming languages

All the non-technical aspects addressed in this manuscript, such as prescribing deadlines and standard syllabi, are equally applicable in other environments. The Packer system for creating reproducible, reusable AMIs is also quite general and can be tailored to install essentially any software, language, or workshop materials. The bookdown package and R markdown software stack that we describe supports more than 40 additional languages besides R ( https://bookdown.org/yihui/rmarkdown/language-engines.html), albeit at a less granular level than the R language support. Jupyter notebooks share some similarities with R markdown and are also quite popular for developing computational workshop materials. The gitbook publishing software ( https://www.gitbook.com/), upon which the bookdown R package is based, is capable of taking the resulting markdown files and producing a book or website.

### Future directions and challenges

Areas of future work include (i) enhancing the quality of individual workshops as well as adding new material, (ii) streamlining the iterative development and build process through continuous integration, and (iii) adopting containers such as Docker as the delivery mechanism rather than the proprietary AMI. We expect that using a social coding paradigm will result in continuous improvement and easy reuse of workshop materials. We plan to adopt a continuous integration pipeline that builds and delivers complete (and versioned) workshop materials. Adjustments to the current build process to leverage parallel processing can speed workshop builds, facilitating the continuous integration approach. Adopting a container technology such as Docker ( https://docker.io) to deliver pre-built workshop materials will increase portability and remove the requirement to use a proprietary system such as Amazon Web Services. Finally, we plan to improve modularity of workshop materials to promote the creation of custom combinations tailored to workshop organizer needs.

## Data availability

All data underlying the results are available as part of the article and no additional source data are required.

## Resource availability

**The workshop book is available from:** https://bioconductor.github.io/BiocWorkshops/.

**All source code and configurations are available at:** https://doi.org/10.18129/B97H03 ^4^.

**License:** CC BY 4.0

**The Amazon Machine Image with the pre-installed workshop materials and required software is available as ID:** ami-bac2c5c5.
