# Supplementary material for: Orchestrating a community-developed computational workshop and accompanying training materials
Source: F1000Res. 2018 Oct 17;7:1656. [Version 1] doi: 10.12688/f1000research.16516.1 (PMC6234736; doi:10.12688/f1000research.16516.1)
Supplement: Peer review history

# Peer review: Referee response for version 1

**DOI:** 10.5256/f1000research.18052.r40382

**Published:** 2018-11-13

**Authors:** Kevin Blighe

**Affiliations:** 1 Biomedical Sciences Research Institute, Ulster University, Coleraine, UK; 2 Department of Cancer Studies and Molecular Medicine, University of Leicester, Leicester, UK

This work addresses issues that are now faced in the biological and medical (biomedical) sciences, namely: reproduciblility (broadly speaking); ease of learning and accessibility to learning materials; maintenance of software, analysis pipelines, and methods; *et cetera*. The authors have a positive track record in this area and are in a good position to address the community in this regard. In particular, the authors focus on workshops as an ideal form of learning, which is certainly true, and make their material available as an e-book and other resources free to the global community.

 Throughout their work, the authors place emphasis on development and testing of code/programs as a team such that these can be later run in any end-user environment seamlessly and without error. These are indeed core fundamentals that need to be embraced in the wider community irrespective of programming language. Of course, other technical factors that are outside of the control of the authors may still hinder workshop progression. Finally, the emphasis on learning in a well-structured and standardised fashion will help future workshops to be successful.

 The text is clear throughout and the authors provide sufficient details such that their efforts can be reproduced by others. Broadly speaking, the push toward standardisation is key to the integration between 'big data' and the biomedical sciences, and here the authors address one component of that.

 Some minor comments:

- On page 3, left column: Could you elaborate on what you mean by ' *Would incorporate some educational best-practices*'? Do you mean a set of pre-existing best practices or just speaking generally? In the same listing, point 7 somewhat overlaps with the second part of point 2 - not a major issue and can be left as is.
- On Page 3, right column: In 'Required technical skills', it is stated ' *The editors exempted one author from the third requirement, but this was for an already well-tested workshop.*' - do you mean the 3rd requirement from the list of requirements for *editors*? There are only 2 requirements in total listed for *authors*.
- Page 4, left column: in 'Deadlines' section, a Gantt chart could help here, but not necessary.
- Page 5, left column: In 'Organization, coordination, and editorial responsibilities', is ' *committee*' the same as ' *multiple editors*'? Could probably clarify this at the start of the list with ' *Except where noted as “committee” or "multiple editors", each task was assigned to a single individual, clarifying responsibilities.*'
- Page 7: it may help to very briefly explain what needs to be done in order to load the AMI for those who have not done this previously

I have read this submission. I believe that I have an appropriate level of expertise to confirm that it is of an acceptable scientific standard.

# Peer review: Referee response for version 1

**DOI:** 10.5256/f1000research.18052.r39598

**Published:** 2018-11-05

**Authors:** Ewy Mathe

**Affiliations:** 1 Department of Biomedical Informatics, Ohio State University, Columbus, OH, USA

The work presented here addresses key topics in the biomedical research community at large, including reproducibility, team coding, installation-independent and error-free code environments. The main goal of the authors was to deliver public resources through published materials through modifiable and raw materials and source codes that can readily be leveraged by participants during and after the Bioconductor training. With this in mind, authors present a generalizable model for producing an open-source workshop in an efficient manner that includes a published work of contributed sessions/education materials that can be used beyond the conference, a guide to best practices in education, an installation-free environment for workshop participants with error-free code. Overall, this approach to creating workshops and educational materials is exemplary for the biomedical research community as it provides public, transparent, reproducible, and free solutions.

 Some minor suggestions that could be included:

- One practical aspect that could be reported is the specific number of editors that was used, particularly given the heavy work load of editors throughout this process. 
- Do participants still have access to an AMI to avoid having to install everything themselves on their own after the workshops?
- There is a small typo in the second sentence of the Discussion “social contracts such a deadlines” should read “social contracts such as deadlines”.
- Under Future directions and challenges, the concept of using a Docker rather than the proprietary AMI is mentioned twice.
- Have authors collected feedback from workshop users, particularly regarding whether they are easily able to use material outside of the conference workshops?

I have read this submission. I believe that I have an appropriate level of expertise to confirm that it is of an acceptable scientific standard.
